# Self-regulation of the posterior cingulate cortex with real-time fMRI neurofeedback augmented mindfulness training in healthy adolescents: A nonrandomized feasibility study

**DOI:** 10.3758/s13415-022-00991-4

**Published:** 2022-03-15

**Authors:** Namik Kirlic, Zsofia P. Cohen, Aki Tsuchiyagaito, Masaya Misaki, Timothy J. McDermott, Robin L. Aupperle, Jennifer L. Stewart, Manpreet K. Singh, Martin P. Paulus, Jerzy Bodurka

**Affiliations:** 1 Laureate Institute for Brain Research, 6655 South Yale Avenue, Tulsa, OK 74136 USA; 2 Department of Psychology, University of Tulsa, Tulsa, OK USA; 3 School of Community Medicine, University of Tulsa, Tulsa, OK USA; 4 Department of Psychiatry and Behavioral Sciences, Stanford University, Palo Alto, CA USA; 5 Stephenson School of Biomedical Engineering, University of Oklahoma, Norman, OK USA

**Keywords:** Mindfulness, Posterior cingulate cortex, Functional magnetic resonance imaging neurofeedback, Self-regulation, Adolescents, Functional connectivity

## Abstract

Mindfulness training (MT) promotes the development of one’s ability to observe and attend to internal and external experiences with objectivity and nonjudgment with evidence to improve psychological well-being. Real-time functional MRI neurofeedback (rtfMRI-nf) is a noninvasive method of modulating activity of a brain region or circuit. The posterior cingulate cortex (PCC) has been hypothesized to be an important hub instantiating a mindful state. This nonrandomized, single-arm study examined the feasibility and tolerability of training typically developing adolescents to self-regulate the posterior cingulate cortex (PCC) using rtfMRI-nf during MT. Thirty-four adolescents (mean age: 15 years; 14 females) completed the neurofeedback augmented mindfulness training task, including Focus-on-Breath (MT), Describe (self-referential thinking), and Rest conditions, across three neurofeedback and two non-neurofeedback runs (Observe, Transfer). Self-report assessments demonstrated the feasibility and tolerability of the task. Neurofeedback runs differed significantly from non-neurofeedback runs for the Focus-on-Breath versus Describe contrast, characterized by decreased activity in the PCC during the Focus-on-Breath condition (z = −2.38 to −6.27). MT neurofeedback neural representation further involved the medial prefrontal cortex, anterior cingulate cortex, dorsolateral prefrontal cortex, posterior insula, hippocampus, and amygdala. State awareness of physical sensations increased following rtfMRI-nf and was maintained at 1-week follow-up (Cohens’ d = 0.69). Findings demonstrate feasibility and tolerability of rtfMRI-nf in healthy adolescents, replicates the role of PCC in MT, and demonstrate a potential neuromodulatory mechanism to leverage and streamline the learning of mindfulness practice. (ClinicalTrials.gov identifier #NCT04053582; August 12, 2019).

**Supplementary Information:**

The online version contains supplementary material available at 10.3758/s13415-022-00991-4.

## Introduction

Mindfulness training (MT) promotes the development of one’s ability to observe and attend to internal experiences, including thoughts, emotions, and behaviors, and thus gain attentional control of the present experience (Shapiro et al., 2006). Specifically, MT can lead to a shift in internal and external perspectives by viewing each moment of experience with objectivity and nonjudgment, thereby improving emotion regulation and cognitive flexibility (Davis & Hayes, 2011; Shapiro et al., 2006). MT has been associated with a number of benefits across a wide developmental spectrum, including reduced symptoms of internalizing disorders (i.e., depression and anxiety), decreased levels of stress reactivity, improved cognitive and social outcomes, and overall physical health (Biegel et al., 2014; Borquist-Conlon et al., 2019; Creswell, 2017; Davis & Hayes, 2011; Khoury et al., 2013; Ortiz & Sibinga, 2017; Zack et al., 2014; Zenner et al., 2014).

MT recruits a distributed network of brain regions (Farb et al., 2007; Hölzel et al., 2007; Tomasino & Fabbro, 2016), particularly the default mode network (DMN) and its key hub, the posterior cingulate cortex (PCC) (Farb et al., 2007; Hölzel et al., 2007; Tang et al., 2015; Tomasino & Fabbro, 2016; Zeidan et al., 2015). The PCC has been shown to facilitate self-referential processing and is predominantly active when one is at rest (Buckner et al., 2008). Specifically, studies have shown that PCC mediates associative, internally generated thought processes during emotional and social events, as well as self-directed cognitions, such as autobiographical memories and future planning (Leech & Sharp, 2014). MT is associated with changes in PCC during self-referential processing, particularly among those who have more extensive experience with the practice (Farb et al., 2007; Grant et al., 2011; Taylor et al., 2011). Moreover, there is a strong correspondence between decreased PCC activity and subjective experience when practicing mindfulness meditation in both novice and expert meditators (Garrison et al., 2013). Finally, MT increases connectivity between the PCC and frontocingulate regions involved in attentional control, as well as with the amygdala and insula, which are involved in emotional and interoceptive awareness (Brewer et al., 2011; Farb et al., 2007). Together, these studies suggest that MT impacts neural activity and functional connectivity of the neurocircuitry involved in self-awareness and attention to internal/external events, as well as circuitry gating emotional responses (Creswell, 2017). Notably, these data implicate the PCC as the prime candidate for the mechanistic understanding of MT.

Numerous studies have demonstrated success in regulation of blood oxygenation level-dependent (BOLD) signal in brain structures via real-time functional magnetic resonance imaging neurofeedback (rtfMRI-nf), including in the amygdala (Posse et al., 2003; Young et al., 2014; Young et al., 2017; Zotev et al., 2011; Zotev et al., 2018), insula (Berman et al., 2013; Caria et al., 2010; Rance et al., 2014), dorsolateral prefrontal cortex (dlPFC) (Sherwood et al., 2016), ventromedial prefrontal cortex (vmPFC) (Ahmad Mayeli et al., 2020; Mayeli et al., 2018), and PCC (Garrison et al., 2013). In adults, successful modulation of BOLD activity within these regions has been in turn associated with reductions in symptoms of depression and anxiety (Young et al., 2017; Zotev et al., 2018), while functional connectivity based fMRI neurofeedback has resulted in improvements in repetitive negative thinking (i.e., rumination) (Tsuchiyagaito et al., 2021).

The role of brain plasticity within the context of MT is still largely unknown, particularly in youth populations. The present study used neurofeedback augmented mindfulness training (NAMT), that is, a combination of rtfMRI-nf with a core strategy of MT (i.e., focusing on breath) to test the feasibility and tolerability of directly engaging and regulating PCC activity in adolescents. rtfMRI-nf has many applications, chief among them the modulation of brain regions thought to play a role in specific psychological processes. When participating in mental or psychological skills training, participants lack the ability to objectively, in real-time assess their performance and the subsequent impact on mental states. This can hinder them from recognizing the effect a particular strategy has on a desired process, thereby diminishing their ability to optimize their performance and outcomes. rtfMRI-nf aids in this process by serving as an objective and moment-by-moment online performance metric of mental training. Specifically, during rtfMRI-nf, participants are presented with BOLD signal from a target region in real time, which is meant to reinforce the learning process of up- or down-regulating the hemodynamic activity in that region (DeCharms, 2008; DeCharms et al., 2005).

Given that the PCC shows promise as a central modulatory target for MT (Brewer & Garrison, 2014), PCC-targeted NAMT may have several beneficial effects on the quality of MT and our understanding of the underlying processes. Specifically, if decreased PCC activity is a product of MT, then effortfully reducing PCC activity could result in better engagement with MT, its improved use, and enhanced outcomes. Furthermore, because rtfMRI-nf may optimize performance of a particular skill, the effects may elucidate underlying neural mechanisms and representations of MT beyond the targeted regions. Adolescent brain neuroplasticity allows for effective attempts at improving learning and performance (Casey et al., 2011), and therefore makes it a critical period to study neural correlates of psychological and behavioral strategies and their optimization. Findings point to significant neurocognitive benefits of MT in the developing brains of youth (Black, 2015), with improvements observed in executive function, metacognition, and behavioral regulation (Lyons & DeLange, 2016). Relative to adults, children and adolescents are better at learning and generalizing knowledge from abstract causal relationships, pay more attention to current evidence rather than prior assumptions, and are engaged more with feedback during learning (Eppinger et al., 2009; Lucas et al., 2014). Thus, adolescence may be an ideal developmental period for rtfMRI-nf based learning. Understanding the neural mechanisms underlying MT and its enhancement when extended to clinical populations could therefore make meaningful strides in reducing the disability associated with mental illness.

The present study serves to examine the feasibility and tolerability of neurofeedback augmented mindfulness training in a sample of typically developing adolescents. As primary feasibility and tolerability outcomes, we evaluated the changes in self-report assessments of number of task-specific outcomes. We predicted that adolescents would exhibit adequate ability to follow task instructions and engage with NAMT, as well as that the overall task would not elicit uncomfortable states. Furthermore, we hypothesized that the neurofeedback signal would correspond with subjective experience of engaging with the NAMT. In addition to feasibility and tolerability outcomes, we tested psychological and neural outcomes as a function of NAMT. Given the lack of a control condition (i.e., sham), we consider these analyses exploratory and interpret them accordingly. First, we examined whether NAMT would result in changes in perceived stress, negative and positive affect, and present-moment mindfulness from before to after NAMT to 1-week follow-up. Next, we evaluated if NAMT elicited changes in PCC activity and if PCC activity during NAMT comodulated other brain areas. Specifically, the primary brain outcome of interest and task manipulation test consisted of change in PCC activity and whether PCC activity differed between baseline during neurofeedback and non-neurofeedback (Observe, Transfer) runs as a result of MT. Finally, we examined changes in whole-brain activation and connectivity.

## Methods

### Participants

Adolescents in the present study comprise the typically-developing sample in a larger ongoing longitudinal study designed to first establish the feasibility and tolerability of NAMT in adolescents and examine the effects of NAMT on neural networks involved in self-referential and emotional processing in adolescents exposed to early life stress. Adolescents were recruited between September of 2019 and June 2021 from the community using flyers, radio and social media advertisements, billboards, and a school-based messaging platform (i.e., PeachJar). A phone screen determined initial eligibility. Remote and in-person visits with adolescents and primary caregivers provided demographic information, medical and psychiatry history, pubertal status, family history of psychiatric illness, and an MRI safety questionnaire. Eligible adolescents were between ages 13 and 17 years at the time of enrollment, had a parent or a legal guardian able to provide consent, were psychiatrically and physically healthy, and were able to validly and safely complete baseline assessments. All races and genders were included. Adolescents had reached puberty at the time of participation as measured with the Pubertal Developmental Scale assessing completion of growth in height, changes in skin, body and facial hair, deepening of the voice, and menstrual cycle (Petersen et al., 1988), and completed by both the parent and adolescent (parent m (SD) = 2.5(0.51); adolescent m(SD) = 2.39(0.38). Adolescents were excluded if diagnosed with a neurological or developmental disorder, were currently being managed for migraines (e.g., daily prophylactic medication), had history of traumatic brain injury, had a lifetime history of psychopathology, were currently using medications with major effects on brain function or blood flow (e.g., acne medication), and/or reported MRI contraindications. As part of the larger study, adolescents completed previous neuroimaging sessions but were naïve to rtfMRI-nf. Parents provided written, informed consent, while adolescents provided written assent for study participation. All study procedures were approved by Western Institutional Review Board and conducted in accordance with the Declaration of Helsinki. The study is registered at the US National Institutes of Health (ClinicalTrials.gov identifier #NCT04053582; August 12, 2019). Thirty-seven adolescents were consented for the present study, with two adolescents withdrawn due to repeated missed appointments following consent procedures, and one participant not having usable data due to technical difficulties, for a total of 34 typically developing adolescents (mean age 15 years, 14 females). According to Desmond and Glover (2002), a sample size of 24 is recommended for typical within-group fMRI experiments, in which inferences regarding the differences in activation between two or more conditions are intended to be made in a single population. Therefore, with 34, we were 97% powered to detect medium size effects (*f* = 0.25) between conditions.

### Experimental procedures

This nonrandomized, single-arm (i.e., no sham control condition) pilot study aimed to examine the feasibility and tolerability of training typically developing adolescents to self-regulate the hemodynamic activity of the PCC. Eligible adolescents completed several self-report measures before and immediately after rtfMRI-nf session, as well as at 1-week follow-up. The Positive and Negative Affective Schedule for Children (PANAS-C) (Hughes & Kendall, 2009) assessed state affect (pre- and post-NAMT), as well as how adolescents felt “during the past week” (1-week follow-up). The PANAS-C subscales have demonstrated adequate internal consistency and moderate convergent and discriminate validity (Hughes & Kendall, 2009). The Perceived Stress Scale (PSS) captured an indication of perceived stress for the same timepoints (Cohen et al., 1983). Finally, the State Mindfulness Scale (SMS) quantified adolescents’ perceived level of attention to and awareness of their present experience (i.e., mind, body, the pleasant/unpleasant/neutral hedonic tones of these objects of awareness, and the qualities thought to characterize mindful awareness) for the same timepoints (Tanay & Bernstein, 2013).

#### Neurofeedback augmented mindfulness training task (NAMT)

Before rtfMRI-nf session, adolescents underwent brief MT. Participants were first given a brief psychoeducational introduction into mindfulness, including that (1) mindfulness refers to paying attention to thoughts, feelings, and physical sensations in the present moment without any judgment, and (2) mindfulness can reduce stress and increase attention. Next, participants were guided through a traditional mindfulness practice focused on the breath (Brewer et al., 2011; Garrison et al., 2013), that is: “*Please pay attention to the physical sensations of your breath where you most strongly feel it. Follow the natural and spontaneous movement of the breath, not trying to change it any way. Just pay attention to it. If you find that your attention wanders to something else, gently bring it back to the physical sensations of the breath*.” Difficulty of performing the task and how mindful they currently feel of their body and mind was assessed. Following practice, adolescents were provided with an opportunity to ask clarification questions. Next, adolescents went into the mock scanner and completed the same mindfulness practice with MRI noises in the background. Adolescents were also given instructions and feedback around minimizing motion while in the scanner. Finally, adolescents were given instructions for the neuroimaging session. Training was manualized to ensure fidelity across participants. MT was delivered by a trained research assistant under the supervision of a licensed clinical psychologist. Training sessions were audio recorded and up to 20% sessions were randomly selected for fidelity ratings by research staff using an unpublished measure developed by NK in consultation with RLA for the purposes of this study. Specifically, on a 3-point Likert scale (0 = no adherence, 1 = adherence identified but weak or flawed, 2 = good adherence) of how closely the research assistant followed the manualized mindfulness training, the fidelity ratings indicate that the manualized training was delivered with satisfactory adherence (M = 95.81%, SD = 3.33%).

**Fig. 1:**
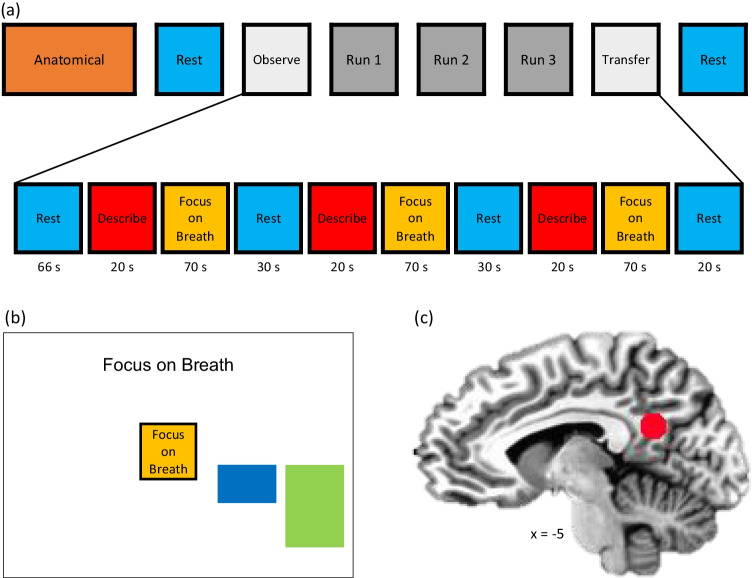
Real-time fMRI neurofeedback paradigm. **a** The experimental protocol consisted of eight fMRI runs, including an anatomical scan, Resting State scan 1 (Rest-1), Observe (OBS), three Neurofeedback runs (NF-1, NF-2, NF-3), a Transfer run (TRS), and Resting State scan 2 (Rest-2). During Rest runs (lasting 6 min), the participants were instructed to clear their minds and not to think about anything in particular while fixating at the display screen. OBS, NF-1, NF-2, NF-3, and TRS runs each lasted 6 minutes and 56 seconds. They started with a 66-s rest block, followed by alternating Focus-on-Breath (Mindfulness Training condition; 70 s), Describe (Active baseline condition; 20 s), and Rest (Baseline condition; 30 s) blocks. During the Focus condition, participants were instructed to pay attention to the physical sensations of their breath, not trying to change it in any way, and if their attention were to wander to something else, to gently bring it back to their breath. In the Describe condition, participants were presented with various adjectives, which they had to mentally categorize as descriptive or not descriptive of them. During the Rest condition, the participants were presented with the cue “Rest” and asked to relax while looking at the display screen. No neurofeedback was provided (no bars displayed) during the Rest and Describe conditions or during the entire OBS and TRS runs. **b** During the Focus condition, participants viewed graphical user interface (GUI) screen with neurofeedback bars (blue) and target bars (green). The participants were told that the blue bar may change with their experience of focusing on the breath, and that their goal to was to make the blue bar match the green bar as often as possible. The target bar remained the same height across neurofeedback runs. **c** Posterior cingulate cortex (PCC, MNI coordinates: x = −5, y = −55, z = 23) was selected as the targeted (ROI, spheres of 7-mm radius) for the real-time fMRI neurofeedback (rtfMRI-nf) training

The neuroimaging session included 8 runs (Fig. 1a), including an anatomical scan, Resting State scan 1 (Rest-1), Observe (OBS), three Neurofeedback runs (NF-1, NF-2, NF-3), Transfer run (TRS), and Resting State scan 2 (Rest-2). During Rest-1 and Rest-2 (6 minutes each), participants were instructed to clear their mind and not think about anything while fixating upon a fixation cross. OBS, NF-1, NF-2, NF-3, and TRS runs each lasted 6 minutes and 56 seconds. Runs started with a 66-s rest block, followed by alternating Describe (Active Control condition without neurofeedback; 20 s), Focus-on-Breath (MT condition with PCC neurofeedback; 70 s), and Rest (Baseline condition; 30 s) blocks. OBS and TRS runs did not involve neurofeedback (no bar displayed) during the Focus-on-Breath condition. The initial long Rest block was required for obtaining enough samples for real-time noise regression analysis (Misaki et al., 2015).

During the Focus-on-Breath condition (Garrison et al., 2013), adolescents were instructed to pay attention to the physical sensations of their breath, not trying to change their breathing in any way, and if their attention were to wander to something else, to gently bring their attention back to their breath (Brewer et al., 2011). To aid in MT, numerous useful strategies were provided prior to scanning, including “Notice the feeling of your belly rising when you breath in, and gently falling when you breath out”; “Notice if it enters and leaves through your nose or your mouth.” In the Describe condition, adolescents were presented with various adjectives, which they had to mentally categorize as descriptive or not descriptive of them for the entire duration the word was displayed on the screen (Kelley et al., 2002). The Describe condition is designed to elicit self-referential thinking, and therefore is thought to be a better comparator to Focus-on-Breath than Rest (Brewer et al., 2011). During the Rest condition, adolescents were presented with the cue “Rest” and asked to relax while looking at the display screen.

During neurofeedback runs, adolescents were told that they would see a bar displayed on the screen, representing the relative brain activity in a particular brain region in real time (Fig. 1b). The instructions further indicated that the bar may change with the experience of focusing on the breath (i.e., the bar may go blue if they are fully concentrating on their breath, and red if their mind wanders elsewhere). The green bar represented the target to attain, and adolescents’ goal was to try and see how much they could make the bar change to blue to match the green bar. The target levels were −0.5%, −0.75%, and −1.0% (percent signal change is relative to the preceding rest block) for the NF-1, NF-2, and NF-3 runs, respectively. Adolescents were told that there might be a 5-s to 6-s delay between their experience and the change in the blue bar. To assess for aspects of feasibility and tolerability, adolescents answered the following questions via a response box and visual analog scale after reach run: (1) How well were you able to follow instructions on the screen? (2) How easy did you find it to focus on your breath? (3) How much did your mind wander while you were asked to focus on your breath? (4) How easy did you find it to mentally decide whether or not the words described you? (5) How easy did you find it to clear your mind while resting? (6) How do you feel right now (1 = perfectly calm, 10 = very anxious)? Two additional questions followed the neurofeedback runs only: (1) How well did the blue bar correspond with your experience of focusing on your breath? (2) How well did the red bar correspond with the experience of your mind wandering elsewhere?

#### Data acquisition

Neuroimaging was performed using a GE MR750 3T MRI scanner with the 8-channel, receive-only head coil. To acquire a T1-weighted anatomical images, 3D magnetization-prepared rapid gradient echo (MPRAGE) pulse sequence accelerated with sensitivity encoding (SENSE) (Pruessmann et al., 1999) was used. The MPRAGE parameters were as follows: FOV/slice thickness = 240/1.2 mm, axial slices per slab = 128, image matrix size = 256 × 256, TR/TE = 5.0/1.9 ms, SENSE acceleration factor R = 2, flip angle = 8°, delay/inversion times TD/TI = 1,400/725 ms, sampling bandwidth = 31.2 kHz, scan time = 5 min 33 s.

For the whole-brain fMRI recording, an accelerated single-shot gradient EPI with SENSE was used. EPI sequence parameters were optimized to maximize sensitivity to BOLD contrast and minimalize image distortion and susceptibility dropouts (Bellgowan et al., 2006; Bodurka et al., 2007). EPI parameters were as follows: FOV/slice = 240/2.9 mm, TR/TE = 2,000/25 ms, SENSE acceleration R = 2, acquisition matrix: 96 × 96, flip angle = 90°, image matrix: 128 × 128, 46 axial slices, voxel volume: 1.9 × 1.9 × 2.9 mm^3^. To allow the fMRI signal to reach a steady state, three EPI volumes (6 s) were added at the beginning of each run and were excluded from data analysis. Physiological pulse oximetry and respiration waveforms were recorded simultaneously with fMRI (with 25-ms sampling interval) using a photoplethysmograph placed on the subject’s finger and a pneumatic respiration belt, respectively. The region of interest (ROI) and rtfMRI-nf target location (spherical ROI, 7-mm radius, [MNI coordinates: x = −5, y = −55, z = 23]; Fig. 1c) was selected based on a meta-analysis investigating functional neuroimaging studies of the DMN (Wang et al., 2020), mindfulness meditation studies, including neurofeedback (Brewer et al., 2011; Garrison et al., 2013), and conducted pilot testing.

#### Real-time fMRI processing

We employed an advanced fMRI real-time processing (RTP) protocol which included: slice-timing correction, motion correction, spatial smoothing with 6 mm-FWHM Gaussian kernel within the brain mask, scaling to a percent change relative to the average for the first 19 TRs (in the initial rest period), and regressing out noise components (Misaki et al., 2015; Misaki & Bodurka, 2021). The noise regressors were six motion parameters, eight RETROICOR (Glover et al., 2000) regressors (4 cardiac and 4 respiration), white matter mean signal, ventricle mean signal, and Legendre polynomial models of slow signal fluctuation. This comprehensive noise reduction was performed in real-time (<400 ms) (Misaki et al., 2015). This fMRI RTP system operates real-time motion tracking, alignment, and motion parameter regression, thus allowing for suppression of head motion effects, and importantly, providing physiological noise correction (RETROICOR) in real time before the PCC based neurofeedback signal computation and visual presentation to the adolescent (Misaki et al., 2015; Wong et al., 2016; Zotev et al., 2014). This ensured that PCC neurofeedback signal reflected the largest possible extent the underlying neuronal activity and does not reflect head motion, heart rate, and/or respiratory motions, all of which overlap with DMN neural activity (Birn et al., 2006; Chang et al., 2009). After real-time noise regression, the fMRI RTP system exports the mean value of the noise-reduced signals for the PCC ROI for each acquired data volume.

The PCC ROI (i.e., neurofeedback target signal) in the MNI space was warped into the individual brain space using the Advanced Normalization Tools (ANTs) software (Avants et al., 2008) (http://stnava.github. io/ANTs/). The neurofeedback stimulus was delivered via custom-developed software using PsychoPy (Peirce et al., 2019). The neurofeedback value was a signal change relative to the baseline obtained by averaging the preceding 30-s long Rest condition. The two initial volumes in the Rest condition were excluded from the baseline calculation to avoid the delayed hemodynamic response effect of the preceding Describe block. The neurofeedback started from the third volume in the Focus block to wait for a hemodynamic response delay. The bar height was updated at every TR as a moving average of the current and up to the two available preceding values to reduce the bar fluctuation (Zotev et al., 2011). The consensus on the reporting and experimental design of clinical and cognitive-behavioral neurofeedback studies (*CRED-nf checklist*) (Ros et al., 2020) is included in the supplementary materials. Sham control condition was not employed.

#### Offline data processing and analysis

AFNI (http://afni.nimh.nih.gov) (Cox, 1996) was used for data image analysis. The first 5 fMRI volumes were discarded and fMRI data preprocessing included despiking, RETROICOR (Glover et al., 2000), respiration volume per time correction (Birn et al., 2008), slice-timing and motion corrections, nonlinear warping to the Montreal Neurological Institute (MNI) template brain with resampling to 2 mm^3^ voxels using the ANTs (Avants et al., 2008), spatial smoothing with a 6-mm FWHM Gaussian kernel, and scaling signal to percent change relative to the mean in each voxel. The general linear model (GLM) analysis was used for independently evaluating the brain response in the OBS, NF-1, NF-2, NF-3, and TRS runs. The design matrix included a modeled response to the Focus-on-Breath block (boxcar function convolved with hemodynamic response function), 12 motion parameters (3 shift and 3 rotation parameters with their temporal derivatives), 3 principal components of the ventricle signal, local white matter average signal (ANATICOR) (Jo et al., 2010), and low-frequency fluctuation (fourth-order Legendre polynomial model). The beta coefficient of the Focus-on-Breath block regressor was extracted to estimate brain activation during each run (OBS, NF-1, NF-2, NF-3, and TRS) and was converted to percent signal change (PSC) for the Focus-on-Breath vs. Describe contrast of the primary brain outcome analysis examining change in the PCC activity as a function of NAMT. Finally, we evaluated the consistency of the Focus-Describe contrast values in PCC using the intraclass correlations (ICC). Because the differences between runs could include the training effect, we evaluated the consistency of the average responses across runs using ICC(3, k) (Shrout & Fleiss, 1979). The ICC was calculated using the “psych” package (Revelle & Revelle, 2017) on R statistical computing environment.

Regarding secondary brain outcomes, a whole-brain group statistic map was created by testing the mean PSC of the Focus-on-Breath vs. Describe contrast during the neurofeedback runs (NF-1, NF-2, NF-3) using AFNI program 3dttest++. The analysis was performed at each voxel, and the statistical map was thresholded with voxel-wise at *p* < 1.0^-6^ for a meaningful separation of clusters, including only clusters consisting of ≥19 contiguous voxels at *p* < 0.005 evaluated with AFNI’s 3dClustSim using an improved spatial autocorrelation function (ACF). Evaluation of participant age and sex did not survive this stringent threshold; therefore, a more lenient yet statistically appropriate threshold was used. Specifically, a voxel-wise threshold of *p* < 0.001 including only clusters consisting of ≥53 contiguous voxels at *p* < 0.005 was used.

Finally, we examined the psychophysiological interaction (PPI) functional connectivity from the PCC to the whole brain in a separate GLM analysis. The average PCC ROI signal time series was multiplied with the Focus-on-Breath block regressor to make a PPI regressor (Di & Biswal, 2017). The design matrix included the PPI regressor, the PCC ROI time series orthogonalized to the PPI regressor in addition to the task block models, and noise regressors as described above. The volumes with >0.3-mm frame-wise displacement were censored in the PPI connectivity analysis. The significance criterion was set with voxel-wise *p* < 1.0^-15^ for a meaningful separation of clusters and cluster-size correction at *p* < 0.005 (cluster size of ≥19 contiguous voxels), as determined with AFNI’s 3dClustSim and ACF. To evaluate the main effect of participant age and sex, a voxel-wise threshold of *p* < 0.001 including only clusters consisting of ≥53 contiguous voxels at *p* < 0.005 was used.

All other statistical analyses were performed using the R statistical package (RCoreTeam, 2013). Descriptive statistics regarding participant characteristics were obtained using the R package “psych” (Revelle, 2015). One-sample *t*-tests examined extracted percent BOLD signal change during each fMRI run against zero. To examine changes in BOLD signal across fMRI runs (OBS, NF-1, NF- 2, NF-3, and TRS), linear mixed effects models (LMEs) were conducted using the “lmer” function in the R package “lme4” (Bates et al., 2007). Fixed effects included each timepoint (OBS, NF-1, NF- 2, NF-3, and TRS), while subject ID number was entered as a random effect. Follow-up pairwise comparisons were conducted using the “glht” function in R package (Hothorn et al., 2008) and corrected for testing all possible pairwise comparisons with the Tukey’s Honestly Significant Difference test. Changes in self-reported measures across different time points (before and immediately after rtfMRI-nf, and at 1-week follow-up) and task-related responses (following each run) were examined with identical LME procedures. Effect sizes were estimated using Cohen’s *f*(J. Cohen, 2013). Finally, Pearson correlation coefficient was used to examine the relationship between PCC BOLD signal during neurofeedback runs and task ratings and self-reported measures (PCC BOLD signal averaged across NF-1, NF-2, NF-3). Bonferroni correction was used to correct for multiple comparisons.

#### Data and code availability statement

The data and ﻿data analysis scripts that support the findings of this study are available on request from the corresponding author after a formal data sharing agreement has been signed. The data are not publicly available due to privacy or ethical restrictions.

## Results

### Participant and task-related behavioral data (feasibility and tolerability of NAMT)

**Fig. 2:**
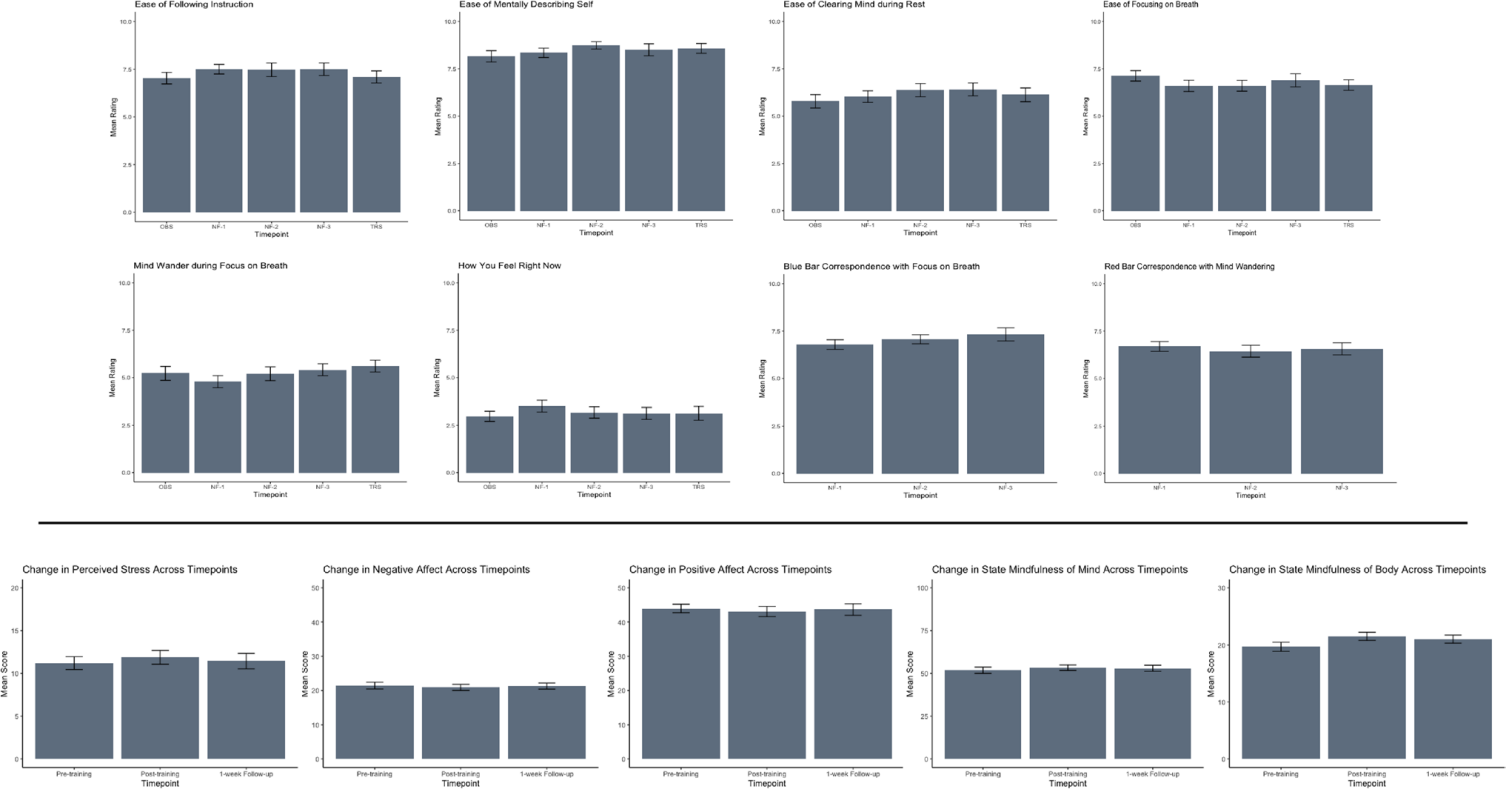
Top Panel: participant reported task ratings for each experimental run: Observe (OBS), Neurofeedback-1 (NF-1), Neurofeedback-2 (NF-2), and Neurofeedback-3 (NF-3), and Transfer (TRS). Task ratings were answered following the completion of each run. Questions included "How well were you able to follow instructions on the screen? How well did the blue bar correspond with your experience of focusing on your breath? How well did the red bar correspond with the experience of your mind wandering elsewhere?" (1 = not at all; 10 = perfectly); "How easy did you find it to mentally decide whether or not the words described you? How easy did you find it to clear your mind while you were resting? How easy did you find it to focus on your breath?" (1 = not easy at all; 10 = very easy); "How much did your mind wander while you were asked to focus on your breath?" (1 = not at all; 10 = all of the time); "How do you feel right now?" (1 = perfectly calm; 10 = very anxious). Tasks questions about the blue and red neurofeedback bars were presented only after completion of NF-1, NF-2, and NF-3. Bottom Panel: participant reported psychological measures across timepoints: pre-training and neurofeedback, immediately post-training and neurofeedback, and at one-week follow-up. Participants evidenced an increase in state mindfulness of the body following NAMT that was maintained at 1-week follow-up. The error bars represent the SE of the mean. Table 1 includes means, standard deviations, and linear mixed effect model results

Of the 34 recruited adolescents, all completed the protocol with no adverse events observed or reported. In general, adolescents reported moderate to high ability to follow instructions on the screen, high ability to mentally decide whether words during the Describe condition described them, moderate ability to clear their mind during the Rest condition, moderate-to-high ability to focus on their breath during the Focus-on-Breath condition, and moderate mind wandering during the Focus-on-Breath condition. Finally, participants reported feeling moderately calm throughout the entire task. The scores on these measures did not differ across fMRI runs (OBS, NF-1, NF- 2, NF-3, and TRS; *p* > 0.05; Table 1; Fig. 2), and there was not an effect of participant age or sex on any scores (*p* > 0.05), except for the effect of sex on the ability to focus on breath [F_(1,29)_ = 5.77, *p* = 0.03], which was higher in males. During neurofeedback, participants reported that the signal bar turning blue corresponded with their experience of focusing on breath to a moderate degree, while the signal bar turning red corresponded moderately with their experience of mind wandering elsewhere. The scores on these measures did not differ across neurofeedback runs (NF-1, NF- 2, NF-3; *p* > 0.05; Table 1; Fig. 2). Similarly, there were no participant age or sex effects (*p* > 0.05). Before NAMT, adolescents reported low levels of perceived stress, low levels of negative affect, high levels of positive affect, and high levels of state mindfulness (body, mind). The scores on perceived stress, positive and negative affect, state mindfulness of the mind did not differ following NAMT or at 1-week follow-up (*p* > 0.05), whereas state mindfulness of the body increased following NAMT and was maintained at 1-week follow-up (*p* < 0.05; Table 1; Fig. 2). No effects of participant age or sex were observed (*p* > 0.05).

**Table 1:** Unadjusted means, standard deviations, effect sizes, and main analyses of change in symptom measures and task ratings across timepoints

Task ratings	Mean	SD	Estimate	SE	t	*p*	Cohen's d
Follow instructions							
OBS	7.03	1.77	--	--	--	--	--
NF-1	7.50	1.46	0.57	0.28	2.05	0.04	0.37
NF-2	7.47	2.10	0.48	0.28	1.71	0.09	0.31
NF-3	7.50	1.91	0.45	0.27	1.63	0.11	0.29
TRS	7.09	1.86	0.09	0.28	0.32	0.75	0.06
Age	--	--	0.09	0.24	0.37	0.71	0.13
Sex	--	--	0.10	0.56	0.17	0.86	0.06
Describe							
OBS	8.16	1.72	--	--	--	--	--
NF-1	8.34	1.45	0.18	0.30	0.61	0.54	0.11
NF-2	8.73	1.14	0.60	0.31	1.94	0.05	0.35
NF-3	8.50	1.85	0.29	0.30	0.98	0.33	0.18
TRS	8.58	1.52	0.39	0.30	1.29	0.20	0.23
Age	--	--	0.18	0.17	1.01	0.32	0.36
Sex	--	--	0.06	0.41	0.14	0.89	0.05
Clear mind							
OBS	5.78	2.06	--	--	--	--	--
NF-1	6.03	1.77	0.38	0.33	1.18	0.24	0.21
NF-2	6.37	2.03	0.63	0.33	1.91	0.06	0.34
NF-3	6.41	1.97	0.64	0.32	1.99	0.05	0.36
TRS	6.12	2.12	0.38	0.32	1.17	0.24	0.21
Age	--	--	0.04	0.24	0.17	0.86	0.06
Sex	--	--	1.04	0.56	1.84	0.08	0.66
Focus on breath							
OBS	7.12	1.60	--	--	--	--	--
NF-1	6.59	1.72	-0.49	0.37	-1.31	0.19	-0.24
NF-2	6.60	1.65	-0.50	0.38	-1.34	0.18	-0.24
NF-3	6.88	2.01	-0.22	0.37	-0.59	0.56	-0.11
TRS	6.64	1.62	-0.44	0.37	-1.21	0.23	-0.22
Age	--	--	-0.16	0.16	-0.99	0.33	-0.36
Sex	--	--	0.86	0.38	2.28	0.03	0.84
Mind wander							
OBS	5.22	2.15	--	--	--	--	--
NF-1	4.78	1.83	-0.51	0.37	-1.38	0.17	-0.25
NF-2	5.20	2.12	-0.06	0.37	-0.15	0.88	-0.03
NF-3	5.41	1.86	0.17	0.36	0.47	0.64	0.08
TRS	5.61	1.85	0.38	0.36	1.06	0.29	0.19
Age	--	--	-0.33	0.22	-1.47	0.15	-0.52
Sex	--	--	-0.21	0.51	-0.40	0.69	-0.14
Blue bar							
NF-1	6.78	1.50	--	--	--	--	--
NF-2	7.07	1.39	0.30	0.32	0.92	0.36	0.24
NF-3	7.32	2.01	0.50	0.31	1.59	0.12	0.41
Age	--	--	0.19	0.19	1.02	0.32	0.36
Sex	--	--	0.91	0.45	2.02	0.05	0.73
Red bar							
NF-1	6.69	1.49	--	--	--	--	--
NF-2	6.43	1.83	-0.23	0.33	-0.68	0.50	-0.17
NF-3	6.56	1.85	-0.19	0.32	-0.58	0.57	-0.15
Age	--	--	0.08	0.21	0.38	0.71	0.13
Sex	--	--	0.16	0.50	0.32	0.75	0.12
Current feeling							
OBS	2.97	1.56	--	--	--	--	--
NF-1	3.50	1.76	0.46	0.31	1.50	0.14	0.27
NF-2	3.17	1.72	0.23	0.31	0.73	0.47	0.13
NF-3	3.12	1.82	0.08	0.30	0.26	0.79	0.05
TRS	3.12	2.12	0.13	0.30	0.44	0.66	0.08
Age	--	--	-0.33	0.21	-1.60	0.12	-0.56
Sex	--	--	0.72	0.48	1.50	0.14	0.54
Symptoms	Mean	SD	Estimate	SE	t	p	Cohen's d
Perceived stress							
T1	11.21	4.40	--	--	--	--	--
T2	11.88	4.77	0.68	0.62	1.10	0.28	0.27
T3	11.44	5.26	0.24	0.62	0.38	0.70	0.09
Age	--	--	-0.14	0.66	-0.21	0.84	-0.07
Sex	--	--	-0.98	1.56	-0.63	0.53	-0.23
Positive affect							
T1	43.97	7.40	--	--	--	--	--
T2	43.09	8.65	-0.88	0.85	1.04	0.30	-0.26
T3	43.67	9.87	-0.22	0.86	-0.26	0.80	-0.06
Age	--	--	2.12	1.18	1.80	0.08	0.65
Sex	--	--	-0.83	2.79	-0.29	0.76	-0.11
Negative affect							
T1	21.45	5.68	--	--	--	--	--
T2	20.91	5.07	-0.42	0.60	-0.71	0.48	-0.17
T3	21.29	5.00	-0.04	0.60	-0.06	0.95	-0.02
Age	--	--	0.59	0.70	0.84	0.41	0.30
Sex	--	--	-2.81	1.66	1.69	0.10	0.61
State mindfulness							
T1	71.47	14.51	--	--	--	--	--
T2	74.91	12.97	3.44	1.80	1.92	0.06	0.47
T3	74.06	14.01	2.59	1.80	1.44	0.15	0.35
Age	--	--	2.83	1.82	1.56	0.13	0.56
Sex	--	--	3.08	4.31	0.72	0.48	0.26
Body							
T1	19.68	4.62	--	--	--	--	--
T2	21.50	4.17	1.82	0.65	2.80	0.01	0.69
T3	21.00	4.18	1.32	0.65	2.03	0.05	0.50
Age	--	--	0.67	0.55	1.20	0.24	0.43
Sex	--	--	1.09	1.31	0.84	0.41	0.30
Mind							
T1	51.79	10.47	--	--	--	--	--
T2	53.41	9.40	1.62	1.28	1.27	0.21	0.31
T3	53.06	10.36	1.27	1.28	0.99	0.33	0.24
Age	--	--	2.16	1.33	1.63	0.11	0.58
Sex	--	--	1.99	3.15	0.63	0.53	0.23

Note. Task ratings were answered following the completion of each run. Questions included "How well were you able to follow instructions on the screen? How well did the blue bar correspond with your experience of focusing on your breath? How well did the red bar correspond with the experience of your mind wandering elsewhere?" (1 = not at all; 10 = perfectly); "How easy did you find it to mentally decide whether or not the words described you? How easy did you find it to clear your mind while you were resting? How easy did you find it to focus on your breath?" (1 = not easy at all; 10 = very easy); "How much did your mind wander while you were asked to focus on your breath?" (1 = not at all; 10 = all of the time); "How do you feel right now?" (1 = perfectly calm; 10 = very anxious). Tasks questions about the blue and red neurofeedback bars were presented only after completion of NF-1, NF-2, and NF-3

*OBS* Observe, *NF* Neurofeedback Run, *TR* Transfer, *T1* Pre-training and pre-MRI, *T2* Post-training and post-MRI, *T3* 1 week follow-up

#### PCC activity

**Fig. 3:**
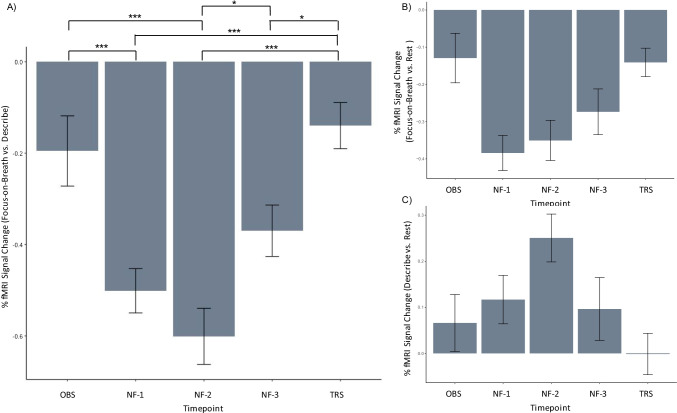
﻿Percent fMRI BOLD signal change for the posterior cingulate cortex (PCC MNI coordinates: x = −5, y = −55, z = 23) for **a** the Focus-on-Breath vs. Describe, **b** Focus-on-Breath only, and **c** Describe only conditions for each experimental run: Observe (OBS), Neurofeedback-1 (NF-1), Neurofeedback-2 (NF-2), and Neurofeedback-3 (NF-3), and Transfer (TRS). The error bars represent the SE of the mean. The results are covaried for participant age and sex. Table 2 includes the mean BOLD signal changes and statistic results for OBS, NF-1, NF2, NF-3, and TRS runs. ^*^p < 0.05; ^**^p < 0.01; ^***^p < 0.001

Figure 3 and Table 2 show the average percent BOLD signal for Focus-on-Breath vs. Describe contrast at the PCC neurofeedback target region. As expected, while covarying for participant age and sex, the contrast in BOLD signal change of Focus-on-Breath vs. Describe was significantly decreased during Focus-on-Breath for all runs [OBS: *t*_(33)_ = −2.54, *p* < 0.05, Cohen’s *d* = −0.44; NF-1: *t*_(33)_ = −10.30, *p* < 0.001, Cohen’s *d* = −1.77; NF-2: *t*_(33)_ = −9.66, *p* < 0.001, Cohen’s *d* = −1.66; NF-3: *t*_(33)_ = −6.56, *p* < 0.001, Cohen’s *d* = −1.12; and TRS: *t*_(33)_ = −2.76, *p* < 0.01, Cohen’s *d* = −0.47]. Findings show (a) decreased PCC activation during the Focus-on-Breath condition relative to Rest [OBS: *t*_(33)_ = −1.94, *p* = 0.06, Cohen’s *d* = −0.33; NF-1: *t*_(33)_ = −8.13, *p* < 0.001, Cohen’s *d* = -1.39; NF- 2: *t*_(33)_ = −6.39, *p* < 0.001, Cohen’s *d* = -1.09; NF-3: *t*_(33)_ = −4.45, *p* < 0.001, Cohen’s *d* = −0.76; and TRS: *t*_(33)_ = −3.70, *p* < 0.001, Cohen’s *d* = −0.63]; and (b) increased PCC activation during the Describe condition relative to Rest [OBS: *t*_(33)_ = 1.07, *p* = 0.30, Cohen’s *d* = 0.18; NF-1: *t*_(33)_ = 2.21, *p* < 0.05, Cohen’s *d* = 0.38; NF- 2: *t*_(33)_ = 4.75, *p* < 0.001, Cohen’s *d* = 0.81; NF-3: *t*_(33)_ = 1.41, *p* = 0.17, Cohen’s *d* = 0.24; and TRS: *t*_(33)_ = −0.03, *p* = 0.97, Cohen’s *d* = −0.01]. There was a main effect of run on the BOLD signal [F_(4,131)_ = 14.24, *p* < 0.001, R^2^ = 0.20] for the Focus-on-Breath vs. Describe contrast, such that neurofeedback runs (NF-1, NF-2, NF-3) differed significantly from the OBS and TRS runs (*p* < 0.05), whereas TRS run did not differ from the OBS run (*p* > 0.05) when corrected for multiple comparisons (Table 3). No main effects of age [F_(1,30)_ = 0.50, *p* = 0.48] or gender [F_(1,31)_ = 0.22, *p* = 0.64] were observed. Figure S1 shows individual adolescents’ PCC responses during the NAMT task. Finally, the consistency of the Focus-Describe contrast as measured by ICC was 0.616 (95% confidence interval, 0.415-0.766), indicating a moderate consistency across participants.

**Table 2:** BOLD signal change (Focus on Breath vs. Describe, Focus, and Describe) for the posterior cingulate cortex region of interest (PCC MNI coordinates: x = −5, y = −55, z = 23) across fMRI runs

Run	Mean	SD	t	p	Cohen’s effect size
Focus-on-Breath					
OBS	-0.20	0.45	-2.54	0.02	-0.44
NF-1	-0.50	0.28	-10.30	0.00	-1.77
NF-2	-0.60	0.36	-9.66	0.00	-1.66
NF-3	-0.37	0.33	-6.56	0.00	-1.12
TR	-0.14	0.29	-2.76	0.01	-0.47
Focus					
OBS	-0.13	0.39	-1.94	0.06	-0.33
NF-1	-0.38	0.28	-8.13	0.00	-1.39
NF-2	-0.35	0.31	-6.39	0.00	-1.10
NF-3	-0.27	0.36	-4.45	0.00	-0.76
TR	0.14	0.22	-3.70	0.00	-0.63
Describe					
OBS	0.07	0.36	1.07	0.30	0.18
NF-1	0.12	0.31	2.22	0.03	0.38
NF-2	0.25	0.30	4.75	0.00	0.82
NF-3	0.10	0.40	1.41	0.17	0.24
TR	0.00	0.26	-0.03	0.98	-0.01

*NF* Neurofeedback, *OBS* Observe, *TR* Transfer

**Table 3:** Posthoc comparisons for Breath vs. Describe conditions contrast for the posterior cingulate cortex (PCC) across experimental runs

Run	Estimate	Std. error	z statistic	*p*
NF-1:OBS	-0.31	0.07	-4.17	<0.001
NF-2:OBS	-0.41	0.07	-5.52	<0.001
NF-3:OBS	-0.17	0.07	-2.38	0.12
TR:OBS	0.06	0.07	0.76	0.94
NF-2:NF-1	-0.10	0.07	-1.39	0.64
NF-3:NF-1	0.13	0.07	1.79	0.38
TR:NF-1	0.36	0.07	4.93	<0.001
NF-3:NF-2	0.23	0.07	3.16	0.01
TR:NF-2	0.46	0.07	6.27	<0.001
TR:NF-3	0.23	0.07	3.14	0.02

*NF* Neurofeedback, *OBS* Observe, *TR* Transfer

Covaried for participant age and sex, lower PCC activity was associated with greater correspondence between the neurofeedback bar and subjective experience of focusing on breath during NF-1 (*p* < 0.01) and NF-2 (*p* < 0.05), but these results did not survive Bonferroni correction for multiple comparisons. PCC activity during neurofeedback runs was not associated with awareness of mind and body, perceived stress, or positive or negative affect. Table 4 presents detailed correlational results between PCC activity and NAMT task ratings and self-report measures.

**Table 4:** Correlations between PCC activity and task ratings and self-report measures

Task measure	PCC NF-1	PCC NF-2	PCC NF-3
Correspondence with NF-1 Bar	-0.45***	-	-
Correspondence with NF-2 Bar	-	-0.44**	-
Correspondence with NF-3 Far	-	-	-0.23
Ease of Focus During NF-1	-0.30*	-	-
Ease of Focus During NF-2	-	-0.14	-
Ease of Focus During NF-3	-	-	-0.16
Self-Report Measure			
SMS Body Pre NAMT	0.10	0.03	0.02
SMS Body Post NAMT	-0.18	-0.12	-0.09
SMS Body 1-week Post NAMT	-0.13	-0.05	0.11
SMS Mind Pre NAMT	0.04	0.04	0.00
SMS Mind Post NAMT	-0.04	-0.06	-0.04
SMS Mind 1-week Post NAMT	-0.06	0.03	-0.07
PSS Pre NAMT	0.13	0.09	0.22
PSS Post NAMT	0.04	0.09	0.09
PSS 1-week Post NAMT	0.00	0.01	0.11
PA Pre NAMT	0.03	0.21	0.18
PA Post NAMT	0.00	0.08	0.21
PA 1-week Post NAMT	0.01	0.23	0.01
NA Pre NAMT	-0.21	-0.08	0.15
NA Post NAMT	-0.12	-0.10	0.22
NA 1-week Post NAMT	-0.05	0.10	0.23

NA, PANAS Negative Affect; NAMT, Neurofeedback-augmented Mindfulness Training; NF, Neurofeedback Run; PA, PANAS Positive Affect; PSS, Perceived Stress Scale; SMS, State Mindfulness Scale

*** *p* < 0.01; ** *p* < 0.05; * *p* < 0.10

#### Whole brain and connectivity results

**Fig. 4:**
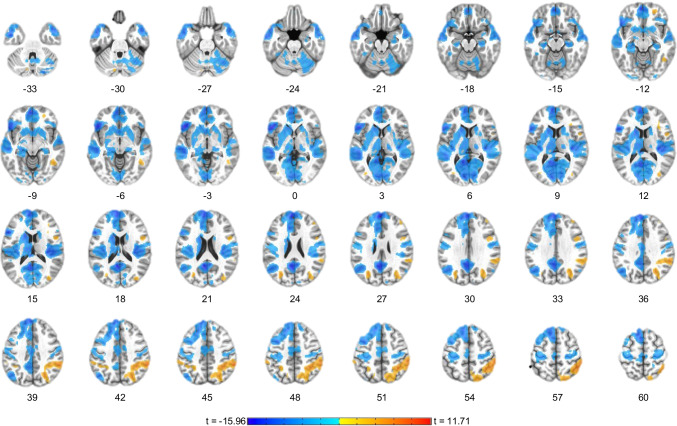
Activation Network for Focus-on-Breath vs. Describe condition. The group fMRI activation analysis for the Focus-on-Breath vs. Describe contrast revealed significant BOLD signal changes in the precuneus, middle orbital gyrus, superior medial gyrus, anterior cingulate cortex, posterior insula, caudate, thalamus, hippocampus, amygdala, superior frontal gyrus, cerebellum, and inferior frontal gyrus. Table 5 shows cluster peak coordinates. The activation maps are projected on the MNI152 standard-space T1-weighted average structural template. *p* < 1.0^-6^, cluster sized corrected *p* < 0.005 (19 voxels). Activation map is covaried for participant age and sex. Left is left

Covaried for participant age and sex, the results show that neurofeedback effects were not isolated to PCC, but further related to deactivations in the precuneus, angular gyrus (AG), mPFC, dorsal ACC, posterior insula, parahippocampal gyrus, hippocampus, amygdala, thalamus, putamen, and caudate nucleus, and increased activations in the supramarginal gyrus, dlPFC inferior parietal lobule (IPL), temporal pole, and inferior frontal gyrus (IFG) among others (Fig. 4; Table 5). The main effect of age was evident in the left inferior temporal gyrus (x = −43, y = −1, z = −31), whereas the main effect of sex was evident in the right supramarginal gyrus (x = 51, y = −41, z = 43).

**Table 5:** Peak coordinates of the clusters from group fMRI analysis for the mean Focus-on-Breath vs. Describe contrast during the neurofeedback runs (NF-1, NF-2, NF-3)

Hemisphere / location	Peak coordinates in MNI			t	Volume (mm3)
L Inferior Frontal Gyrus	-49	25	1	-15.96	44173
R SupraMarginal Gyrus	61	-43	43	11.71	3492
L Angular Gyrus	-49	-65	35	-10.9	1307
R Thalamus	17	-21	5	-9.65	646
R Inferior Frontal Gyrus	51	5	17	7.74	278
R Superior Frontal Gyrus	15	35	57	-7.38	233
L Middle Occipital Gyrus	-29	-71	25	8.25	207
L Inferior Parietal Lobule	-27	-49	47	6.79	178
R Inferior Temporal Gyrus	45	-55	-9	7.1	141
L Inferior Parietal Lobule	-63	-37	49	7.59	128
L Fusiform Gyrus	-35	-43	-23	-7.02	87
L Inferior Occipital Gyrus	-33	-91	-9	-5.97	81
R Superior Parietal Lobule	19	-49	69	-5.96	73
R Middle Orbital Gyrus	25	51	-11	7.59	70
R Inferior Frontal Gyrus	35	31	11	6.14	55
R Middle Frontal Gyrus	43	43	27	6.18	54
R Angular Gyrus	53	-65	31	-6.31	39
L Cerebellum	-13	-77	-33	7.21	37
L Middle Occipital Gyrus	-35	-79	3	6.75	29
L Inferior Occipital Gyrus	27	-93	-11	-6.27	28
L Precuneus	-11	-73	47	5.46	20

Note. The x, y, z coordinates indicate distance in millimeters from the anterior commissure in three dimensions: x, right to left; y, anterior to posterior; z, dorsal to ventral with positive values indicating right, anterior or dorsal and negative values left, posterior or ventral, respectively. The number of voxels in each cluster reflects contiguous voxels in which *p* < 0.000001 after applying appropriate corrections for multiple comparisons. All coordinates reported according to MNI space. L, Left; NF, Neurofeedback Run; R, Right

**Fig. 5:**
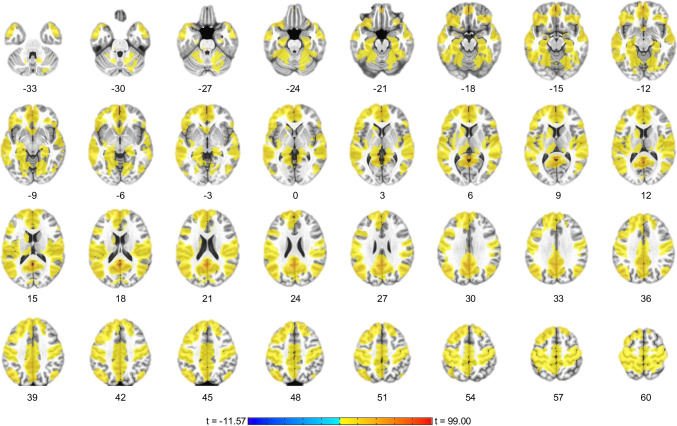
﻿Real-time neurofeedback functional connectivity correlation map. The real-time neurofeedback from the PCC target during Focus blocks was used as a regressor in fMRI GLM analysis across neurofeedback (NF-1, NF-2, NF3) runs. Strong correlations are evident between the real-time feedback and other brain regions within the DMN, including mPFC, precuneus, angular gyrus, and parietal lobule, as well as inferior frontal gyrus, middle frontal gyrus, cingulate cortex, insula, caudate, thalamus hippocampus, amygdala, and cerebellum. Table 6 shows cluster peak coordinates. The activation maps are projected on the MNI152 standard-space T1-weighted average structural template. *p* < 1.0^-15^, cluster sized corrected *p* < 0.005 (19 voxels). Activation map is covaried for participant age and sex. Left is left

We also investigated brain regions that may be associated with and influenced by the average signal change in the PCC target during neurofeedback conditions and thereby form a PCC-MT network. Covaried for participant age and sex, rtfMRI-nf PCC target average signal for the Focus-on-Breath condition was associated with many brain regions, included a large cluster centered on the precuneus and including mPFC, rostral and dorsal ACC, amygdala, parahippocampal gyrus, hippocampus, MFG, IFG, AG, parietal lobule, insula, thalamus, putamen, and caudate nucleus, as well as the brainstem (Fig. 5; Table 6). The main effect of age was evident in the bilateral postcentral gyrus (x = 17, y = −39, z = 63; x = −29, y = −37, z = 51), left precentral gyrus (x = −57, y = −1, z = 31), and right superior frontal gyrus (x = 21, y = 13, z = 57), whereas the main effect of sex was evident in the left middle temporal (x = −59, y = −59, z = 19) and precentral gyri (x = −49, y = 19, z = −17) and left temporal pole (x = −37, y = 11, z = 43).

**Table 6:** Peak coordinates from the whole brain PPC ROI real-time neurofeedback signal functional connectivity analysis during neurofeedback runs (NF-1, NF-2, NF-3)

Hemisphere / location	Peak coordinates in MNI			t	Volume (mm3)
L Precuneus	-5	-55	19	99	79775
Brainstem	-13	-31	33	10.41	22
L Olfactory Cortex	-13	9	-17	10.99	21

Note. The x, y, z coordinates indicate distance in millimeters from the anterior commissure in three dimensions: x, right to left; y, anterior to posterior; z, dorsal to ventral with positive values indicating right, anterior or dorsal and negative values left, posterior or ventral, respectively. The number of voxels in each cluster reflects contiguous voxels in which *p* < 1.0-15 after applying appropriate corrections for multiple comparisons. All coordinates reported according to MNI space. L, Left; NF, Neurofeedback Run; R, Right

## Discussion

The present study aimed to (1) examine the feasibility and tolerability of self-regulation of the PCC via rtfMRI-nf and (2) to determine the relationship between PCC activity and other brain areas during rtfMRI-nf. First, rtfMRI-nf was a feasible and acceptable approach to self-regulation of brain activity in typically developing adolescents, as indicated by full-sample completion rate, no reported adverse events, no worsening of state affect, and overall positive ratings during NAMT. Second, NAMT was associated with significantly lower activity in the PCC during MT compared with self-referential processing (Focus-on-Breath vs. Describe) in adolescents. Moreover, decreased PCC activity was significantly more pronounced during MT when presented with rtfMRI-nf compared to without rtfMRI-nf, indicating that the effect of rtfMRI-nf on PCC activity was above and beyond MT alone. Although the lack of neurofeedback control condition precludes us from inferring causality, this suggests that PCC rtfMRI-nf may be useful in enhancing the effects of MT on neural mechanisms involved in this process. Nevertheless, there was no significant difference in the PCC activity between OBS and TRS, which indicates that the learning during rtfMRI-nf may not have optimally generalized to non-rtfMRI-nf conditions in the PCC. During rtfMRI-nf training, decreased PCC activity during MT condition was associated with increased correspondence between the rtfMRI-nf bar and focus on the breath. Finally, whole-brain analysis showed deactivations in several regions of the DMN and SN. Fourth, rtfMRI-nf PCC signal during MT was strongly correlated with activity in the regions of mPFC, dorsal ACC (dACC), posterior insula, hippocampus, and amygdala among other regions.

The self-report task rating data suggest that healthy adolescents in our study understood and were able to complete the NAMT task without difficulty. Overall, adolescents did not find the task to be distressing or uncomfortable. Moreover, they expressed moderate to high ability to engage with each specific task condition, including the ability to clear their mind during Rest, engage in self-referential thinking during Describe, and pay attention to physical sensations of their breath during Focus-on-Breath conditions. Notably, adolescents reported mind-wandering during the Focus-on-Breath condition, signifying that focusing on physical breath sensations required sustained attention and effort. These findings are consistent with previous reports in novice adult meditators (Garrison et al., 2013) and with the well-understood notion that it is typical for the mind to wander during MT. Adolescent males in this sample reported greater ability to focus on breath, but this effect was not evident in changes in PCC activity. Nevertheless, adolescents had moderate to high correspondence between their experience of breath focus, mind-wandering, and the height and color of the feedback bar. Taken together, these data suggest that real-time neurofeedback MT is a feasible and tolerable approach to use with adolescents.

It is somewhat surprising that the self-reported ease of breath focus and mind-wandering did not differ between neurofeedback and non-neurofeedback (i.e., OBS and TRS) runs, especially because there were such differences in PCC activity (see below). The discrepancy between subjective experience and objective measures of behavior has been previously reported (Dang et al., 2020). This can occur particularly in cases of limited variability in self-report measurement, as is the case in this healthy sample. Furthermore, it is possible that participants did not observe significant changes in their experience of focus on breath and mind-wandering given that these were measured after each functional run rather than after each Focus-on-Breath trial. Finally, although PCC activity did not relate to one’s perception of mind-wandering and focus on breath, the relative difference in PCC activity during mindfulness practice as compared to rest and self-referential thinking, and to a greater extent during neurofeedback trials, points to its direct involvement in MT.

The results evidenced a relationship between neurofeedback training and PCC activity. Relative to Describe, the Focus-on-Breath (i.e., MT) condition while receiving real time information about PCC activity related to significant and sustained deactivation of the PCC across neurofeedback runs. It is noteworthy that similar PCC findings were observed during non-neurofeedback runs, albeit this deactivation being significantly smaller in magnitude. This provides evidence that, relative to self-referential thinking, mindfulness practice relates to attenuation of the PCC activity, and importantly, that this effect may be further enhanced by augmentation of mindfulness practice via PCC-targeted neurofeedback. The same pattern was observed for the Focus-on-Breath relative to Rest contrast, suggesting that MT and its augmentation with neurofeedback may have a unique effect on PCC and related neurocircuitry. While promising, these within-subject results require confirmation by between-subject evaluations using supplementary control conditions such as sham neurofeedback. The sham condition would ensure that the neurofeedback training effects are not induced by various factors other than the feedback of one’s own brain activation, including motivation, perception of success with a rewarding feedback signal, or placebo or expectation effect.

Although neurofeedback runs evidenced significantly lower PCC activity relative to non-neurofeedback runs, we did not observe a significant progressive learning effect of neurofeedback on PCC activity as has been reported by previous studies across different brain regions, including the ACC, anterior insula, and amygdala (Caria et al., 2007; DeCharms et al., 2005; Zotev et al., 2011). The observed effect of neurofeedback also did not generalize to the TRS run as it had in other studies (Caria et al., 2007; Zotev et al., 2011), as evidenced by nonsignificant difference in PCC activity from the OBS run. There are several potential explanations for these findings. Although this study is not the first study to target PCC with mindfulness neurofeedback (Garrison et al., 2013), it is the first to focus on typically developing adolescents, and the first to use a post-neurofeedback run (e.g., TRS) to examine sustained effects on the PCC. One possibility is that the learning effects are less pronounced in younger populations due to developmentally appropriate limits on the ability to hold attention and increased fatigue experienced during neuroimaging studies. It also may be the case that NAMT requires fewer neurofeedback runs but greater number of training sessions in order to observe sustained neuronal changes in PCC activity in the absence of neurofeedback augmentation. Nevertheless, if confirmed against neurofeedback control conditions, the effortful modulation of PCC with neurofeedback training may be relevant for the development of novel therapeutic approaches to treat psychiatric disorders.

In these typically developing adolescents, neurofeedback augmented MT was associated with an increase in self-reported awareness of physical sensations, which was maintained at 1-week follow-up. There were no changes in awareness of thoughts and emotions, perceived stress, or positive and negative affect as a function of the task. However, scores within normal range for these measures may explain a lack of changes following rtfMRI-nf training. Nevertheless, this supports further investigation of the role of PCC in mindfulness practice and its promise in exerting effects on psychological measures.

The findings also provided evidence for changes on the whole-brain and connectivity levels as a function of NAMT. Indeed, the whole-brain voxel-wise analysis showed that self-regulation of the PCC while engaging in MT was not isolated to PCC and subsequently related to deactivations in a broad network of regions, including the areas of the DMN (e.g., mPFC, angular gyrus, hippocampus, and IPL) and SN (e.g., dACC, insular cortex, caudate, and thalamus). Analysis of functional connectivity for the PCC-MT network during rtfMRI-nf similarly revealed a positive correlation with a number of regions within the DMN and SN, including the bilateral amygdala. These results are consistent with anatomical studies that show strong connections between PCC and the medial temporal lobe (i.e., hippocampus and parahippocampal gyrus), the IPL, and PFC/ACC (Buckner et al., 2008). Although the amygdala does not have strong direct connections with PCC (Robinson et al., 2010; Stein et al., 2007), previous studies show that the two regions share an important functional relationship as assessed by task- and contrast-specific data across emotional studies (Bzdok et al., 2013; Robinson et al., 2010). This connection is likely achieved by alternative pathways between the two regions: one via the extensive projections from amygdala to the orbitofrontal cortex (OFC) and vmPFC (Carmichael & Price, 1995), and the other from the amygdala to the retrosplenial cortex and parahippocampal gyrus, both of which have their own extensive connections with PCC (Stefanacci et al., 1996; Stein et al., 2007). Lastly, the amygdala and insula have extensive interconnections, and the insula is in turn strongly connected with the PCC and lateral OFC (Mesulam & Mufson, 1982). Our findings also are consistent with a meta-analysis that reported widespread brain activations across rtfMRI-nf studies, including in the insula, striatum, ACC, dlPFC, ventrolateral prefrontal cortex (vlPFC), temporoparietal area, and temporooccipital junction, which the authors referred to as the self-regulation network (Emmert et al., 2016). Taken together, these findings provide support that MT engages brain networks involved in self-referential, emotional, and interoceptive processing. Future research employing control conditions such as sham neurofeedback is needed to confirm the unique effect of neurofeedback on the whole-brain and connectivity changes.

Internalizing disorders (depression and anxiety), which most often emerge in adolescence (Kessler et al., 2007), are characterized by dysregulation of self-referential thinking (Clark, 1999; Nolen-Hoeksema et al., 2008), as well as aberrant function in the cortical midline regions, including the PCC and mPFC (Cooney et al., 2010; Drevets et al., 2008; Jacob et al., 2020; Koolschijn et al., 2009; Nejad et al., 2013; Renner et al., 2015; Servaas et al., 2014; Steinfurth et al., 2017). MT promotes a shift in the internal experience away from negative self-focus and personal identification to present-centered experiential awareness. Therefore, validated PCC-neurofeedback could potentially be a feasible and effective approach for enhancing the effect of MT on regulation of self-referential thinking and other mindfulness-retaled constructs in order to improve outcomes. Future longitudinal studies will be able to test these hypotheses in at-risk and clinical samples of adolescents.

### Limitations

First, although we were well powered to examine differences in activation across experimental conditions in a single population, the sample size of the present study was relatively small. Future studies will need to include larger and more diverse samples across gender, age, and race and ethnicity, not only to confirm these results but also to examine their generalizability and evaluate effects of potential mediating and moderating factors. Second, the present study did not employ a sham condition against which the effects of rtfMRI-nf on PCC activity and functional connectivity could be evaluated. Although we were able to test differences between neurofeedback and non-neurofeedback MT via OBS and TRS runs, the causal effect of PCC neurofeedback cannot be established by this study. Future studies employing multiple levels of control will be able to establish whether these effects are specific to rtfMRI-nf. Third, although previous research provides strong theoretical rationale for targeting PCC with MT and rtfMRI-nf, other regions of the DMN or SN may play an important role in these processes and warrant exploration. Finally, our study included typically developing adolescents only, who reported low levels of perceived stress and negative affect, and relatively high levels of positive affect and state mindfulness. This in part may explain a lack of change in some of the self-reported measures as a result of NAMT. Future studies with clinical populations will be able to explore the effect of NAMT on these and symptom measures.

## Conclusions

Notwithstanding the described limitations, the present study provides initial evidence for feasibility and tolerability of neurofeedback augmented mindfulness training in adolescents. In addition, findings show initial evidence for adolescents’ capacity to decrease PCC neural activity with rtfMRI-nf during mindfulness training. Finally, the present study extends previous findings by showing that the effect of PCC rtfMRI-nf during mindfulness training is not isolated to PCC alone, but rather encompasses a broad network of regions within the DMN and SN involved in self-referential, emotional, and interoceptive processing. Therefore, the neurofeedback augmented MT, that is, combined standard MT with rtfMRI-nf (NAMT), potentially presents a neuromodulatory mechanism to leverage and streamline the learning of mindfulness practice to regulate self-referential processing in typically developing adolescents and apply them to those at risk for or already showing symptoms of internalizing psychopathology.

## Supplementary information

ESM 1 (DOCX 867 kb)

## Data Availability

None of the data or materials for the experiments reported here is available, and none of the experiments was preregistered. The data and ﻿data analysis scripts that support the findings of this study are available on request from the corresponding author after a formal data sharing agreement has been signed. The data are not publicly available due to privacy or ethical restrictions.
