# Supplementary material for: Self-regulation of the posterior cingulate cortex with real-time fMRI neurofeedback augmented mindfulness training in healthy adolescents: A nonrandomized feasibility study
Source: Cogn Affect Behav Neurosci. 2022 Mar 15;22(4):849–67. doi: 10.3758/s13415-022-00991-4 (PMC9293874; doi:10.3758/s13415-022-00991-4)
Supplement: Supplementary file 1 — (DOCX 867 kb) [file 13415_2022_991_MOESM1_ESM.docx]

**Self-Regulation of the Posterior Cingulate Cortex with Real-Time fMRI Neurofeedback Augmented Mindfulness Training in Healthy Adolescents**

Namik Kirlic, PhD^1^; Zsofia P. Cohen, BS^1^; Aki Tsuchiyagaito, PhD^1^; Masaya Misaki, PhD^1^; Timothy J. McDermott, MA^1,2^; Robin L. Aupperle, PhD^1,3^; Jennifer L. Stewart PhD^1,3^; Manpreet K. Singh, MD, MS^4^; Martin P. Paulus^1,3^, MD; Jerzy Bodurka, PhD^1,5^

^1^Laureate Institute for Brain Research, Tulsa, OK

^2^Department of Psychology, University of Tulsa, Tulsa, Oklahoma

^3^School of Community Medicine, University of Tulsa, Tulsa, Oklahoma

^4^Department of Psychiatry and Behavioral Sciences, Stanford University, Palo Alto, California

^5﻿^Stephenson School of Biomedical Engineering, University of Oklahoma, Norman, Oklahoma

**Address correspondence to**: Namik Kirlic, PhD; Associate Investigator; Laureate Institute for Brain Research; 6655 South Yale Ave; Tulsa, OK 74136; Phone: 918-502-5747; Email: nkirlic@laureateinstitute.org

**Supplementary Materials**

Overview

1. Supplementary Tables
   1. Tables S1. Consensus on the Reporting and Experimental Design of clinical and cognitive-behavioural Neurofeedback studies (CRED-nf) best practices checklist
2. Supplementary Figures
   1. Figure S1. Plots of individual adolescents’ posterior cingulate cortex (PCC) responses for the Focus-on-Breath vs. Describe and Focus-on-Breath contrast across task runs.
3. **Supplementary Tables**

Table 1. Consensus on the Reporting and Experimental Design of clinical and cognitive-behavioural Neurofeedback studies (CRED-nf) best practices checklist 2020* (an online tool to complete this checklist is available at [[rtfin.org/CREDnf](http://www.rtfin.org/CREDnf)](http://www.rtfin.org/CREDnf)).

| **Domain** | **Item #** | **Checklist item** | **Reported on page #** |
| --- | --- | --- | --- |
| **Pre-experiment** | | | |
|  | 1a | Pre-register experimental protocol and planned analyses | n/a |
|  | 1b | Justify sample size | 7 |
| **Control groups** | | | |
|  | 2a | Employ control group(s) or control condition(s) | 8-10 |
|  | 2b | When leveraging experimental designs where a double-blind is possible, use a double-blind | n/a |
|  | 2c | Blind those who rate the outcomes, and when possible, the statisticians involved | n/a |
|  | 2d | Examine to what extent participants and experimenters remain blinded | n/a |
|  | 2e | In clinical efficacy studies, employ a standard-of-care intervention group as a benchmark for improvement | n/a |
| **Control measures** | | | |
|  | 3a | Collect data on psychosocial factors | 8 |
|  | 3b | Report whether participants were provided with a strategy | 8-10 |
|  | 3c | Report the strategies participants used | n/a |
|  | 3d | Report methods used for online-data processing and artifact correction | 11-12 |
|  | 3e | Report condition and group effects for artifacts | n/a |
| **Feedback specifications** | | | |
|  | 4a | Report how the online-feature extraction was defined | 11-12 |
|  | 4b | Report and justify the reinforcement schedule | 9-10 |
|  | 4c | Report the feedback modality and content | 10 |
|  | 4d | Collect and report all brain activity variable(s) and/or contrasts used for feedback, as displayed to experimental participants | 11-12 |
|  | 4e | Report the hardware and software used | 9-14 |
| **Outcome measures** | | | |
| Brain | 5a | Report neurofeedback regulation success based on the feedback signal | 15 |
|  | 5b | Plot within-session and between-session regulation blocks of feedback variable(s), as well as pre-to-post resting baselines or contrasts | 32-33, 39 |
|  | 5c | Statistically compare the experimental condition/group to the control condition(s)/group(s) (not only each group to baseline measures) | 15-16 |
| Behaviour | 6a | Include measures of clinical or behavioural significance, defined a priori, and describe whether they were reached | 9; 15-16 |
|  | 6b | Run correlational analyses between regulation success and behavioural outcomes | 16, 34 |
| **Data storage** | | |  |
|  | 7a | Upload all materials, analysis scripts, code, and raw data used for analyses, as well as final values, to an open access data repository, when feasible | n/a |

Note: Darker shaded boxes represent *Essential* checklist items; lightly shaded boxes represent *Encouraged* checklist items. We recommend using this checklist in conjunction with the standardized CRED-nf online tool ([rtfin.org/CREDnf](http://www.rtfin.org/CREDnf)) and the CRED-nf article, which explains the motivation behind this checklist and provides details regarding many of the checklist items.

1. **Supplementary Figures**

**Figure S1**. Figure S1. Plots of individual subjects’ posterior cingulate cortex (PCC) responses for the Focus-on-Breath vs. Describe and Focus-on-Breath contrast across task runs. Note: OBS, Observe; NF, Neurofeedback; TRS, Transfer.
